# Atypical Teratoid/Rhabdoid Tumor of the Sellar Region in an Elderly Patient: A Case Report and Literature Review

**DOI:** 10.7759/cureus.95703

**Published:** 2025-10-29

**Authors:** Jessica Thomas, Roshan Afshan, Precious Idogun, Waqqas Tai, Daniel Ezekwudo, Joseph Fullmer, Ishmael Jaiyesimi

**Affiliations:** 1 Hematology and Oncology, Corewell Health William Beaumont University Hospital, Royal Oak, USA; 2 Internal Medicine, Detroit Medical Center/Wayne State University/Sinai Grace Hospital, Detroit, USA; 3 Pathology, Corewell Health William Beaumont University Hospital, Royal Oak, USA

**Keywords:** atypical teratoid/rhabdoid tumor, concurrent chemoradiation therapy, gross total resection (gtr), sellar atypical teratoid/rhabdoid tumor, systemic chemotherapy

## Abstract

Atypical teratoid/rhabdoid tumor (ATRT) is a rare and highly malignant central nervous system (CNS) neoplasm, most commonly diagnosed in children and only infrequently reported in older adults. Classified as a World Health Organization (WHO) grade IV tumor, ATRT carries a poor prognosis and typically requires aggressive, multimodal treatment. Management in elderly patients is particularly challenging due to comorbidities, a lack of evidence-based guidelines, and heightened risk of treatment-related toxicity.

We report the case of a 75-year-old woman with hypertension and hyperlipidemia who presented with progressive dizziness, headache, and blurry vision. Imaging revealed a pituitary macroadenoma compressing the optic chiasm. Following transsphenoidal hypophysectomy, histopathology unexpectedly confirmed ATRT, CNS WHO grade IV. The patient initiated craniospinal radiation and systemic chemotherapy; however, her course was complicated by severe thrombocytopenia, critical illness myopathy, and persistent encephalopathy attributed to prolonged steroid use and metabolic derangements. Interval imaging demonstrated stable disease with reduced mass effect on the optic chiasm. Due to poor treatment tolerance, radiation therapy was discontinued after 26 sessions, and she was discharged to rehabilitation with plans for continued chemotherapy. Unfortunately, her condition further deteriorated due to treatment-related complications, and she ultimately passed away under hospice care.

This case underscores the diagnostic and therapeutic challenges of ATRT in older adults, a population for whom standard pediatric-based regimens may be excessively toxic. It highlights the need for individualized, patient-centered treatment strategies that prioritize disease control, treatment tolerance, functional status, and quality of life.

## Introduction

Atypical teratoid/rhabdoid tumor (ATRT) is a rare and highly aggressive embryonal tumor of the central nervous system (CNS), classified by the World Health Organization (WHO) as a grade IV neoplasm. Although it accounts for only a small fraction of primary CNS tumors overall, it is a significant diagnostic consideration in pediatric neuro-oncology. ATRT is predominantly diagnosed in children under three years of age, while adult presentations are exceedingly rare, and reports in elderly patients remain exceptionally limited [1]. To date, fewer than 100 adult cases have been described in the literature, and only a small subset involve patients over 70 years of age, underscoring the exceptional rarity of this presentation [1]. In children, ATRT most commonly arises in the posterior fossa, whereas adult cases more frequently occur in the cerebral hemispheres or, less commonly, the sellar region [2,3]. In older adults, lesions may clinically and radiographically mimic more prevalent pathologies, such as pituitary macroadenoma, leading to potential delays in diagnosis and treatment.

Molecularly, ATRTs are now classified into three epigenetic subgroups based on deoxyribonucleic acid (DNA) methylation profiling: ATRT-tyrosinase (ATRT-TYR), ATRT-sonic hedgehog (ATRT-SHH), and ATRT-myelocytomatosis (ATRT-MYC) [4]. While pediatric ATRTs span all three groups, adult sellar ATRTs almost exclusively belong to the ATRT-MYC subgroup, which is associated with poor prognosis and more invasive clinical behavior [5]. Unlike pediatric cases, which frequently harbor homozygous SMARCB1 deletions, adult tumors may instead exhibit compound heterozygous mutations, further supporting their distinction as a separate molecular entity [6,7].

Histopathologically, ATRT is characterized by rhabdoid tumor cells and the biallelic inactivation of the SMARCB1 (INI-1) gene, most often confirmed via loss of nuclear INI-1 expression on immunohistochemistry (IHC) [8]. A high Ki-67 proliferation index and vimentin positivity are commonly observed, reflecting its aggressive growth kinetics. Rare cases exhibit the inactivation of SMARCA4 (BRG1), especially in tumors with retained INI-1 expression [8]. In adult-onset ATRT, the proliferative index is often markedly elevated (Ki-67 >50%), and tumors may harbor complex karyotypes, underscoring a uniquely aggressive biological profile [9].

Diagnosis in older adults is particularly challenging due to the tumor's rarity and nonspecific presentation. Magnetic resonance imaging (MRI) of the brain and spine, along with cerebrospinal fluid (CSF) analysis, is recommended to assess for leptomeningeal dissemination [2]. Red flags such as rapid growth, cavernous sinus invasion, or heterogeneous enhancement should prompt the early consideration of ATRT in the differential diagnosis of sellar masses. Definitive diagnosis requires surgical sampling with immunohistochemical staining for SMARCB1/INI-1 or SMARCA4/BRG1 protein loss [10], followed by confirmatory DNA methylation profiling when available, typically completed within one to two weeks. This integrated diagnostic pathway ensures the timely distinction of ATRT from more common lesions such as pituitary macroadenoma or meningioma.

Treatment approaches for ATRT differ markedly between pediatric and adult populations. In children, standard paradigms typically include maximal safe resection followed by craniospinal irradiation, often delivered with proton therapy to minimize long-term normal tissue toxicity, and intensive multi-agent chemotherapy, occasionally incorporating high-dose regimens with autologous stem cell rescue [2]. In contrast, elderly patients face substantial evidence gaps and tolerability constraints. Advanced age, medical comorbidities, diminished bone marrow reserve, and heightened risk of neurotoxicity often preclude the use of aggressive pediatric-based protocols, requiring the careful recalibration of radiation dose, field coverage, and chemotherapy regimens to balance disease control with treatment burden and quality of life.

Prognosis remains dismal across all age groups, though adult and elderly patients often experience worse outcomes due to delayed recognition, comorbidities, and limited tolerability of aggressive therapy. Gross total resection (GTR) is associated with improved survival; in one series, the median overall survival (OS) was 28 months after GTR versus 17 months after subtotal resection [1,9]. A high Ki-67 proliferation index (≥35%) strongly correlates with shorter OS [1,9]. Additional adverse prognostic factors include germline SMARCB1 mutations, MYC-subgroup classification, and metastatic disease at diagnosis, particularly leptomeningeal or pulmonary dissemination [5,11].

Adult-onset ATRTs often deviate from classical pediatric patterns in both anatomic distribution and clinical behavior [12]. Sellar tumors may cause cranial neuropathies and invade adjacent structures including the cavernous sinus. Unlike pediatric cases, adult sellar ATRTs may demonstrate hematogenous spread, with lung metastases reported in some cases [13]. Leptomeningeal involvement remains frequent and carries an ominous prognosis [3]. Despite multimodal treatment strategies involving surgery, radiation, and chemotherapy, the median survival for adult patients typically does not exceed 20 months and is often considerably shorter in the elderly [14].

In managing ATRT in older adults, clinicians must weigh multiple competing priorities: preservation of visual function and endocrine homeostasis, maintenance of neurocognitive performance and functional independence, OS, and quality of life. These patient-centered outcomes must be balanced against substantial treatment-related burdens, including prolonged hospitalizations, transfusion requirements, management of chemoradiation toxicity, and the cumulative impact of extended craniospinal irradiation. Individualized care plans and multidisciplinary collaboration, involving neuro-oncology, radiation oncology, endocrinology, geriatrics, and palliative care, are essential in this population.

This case report illustrates the diagnostic and therapeutic complexities of sellar ATRT in an elderly patient. It underscores the critical need for heightened clinical awareness, early incorporation of advanced diagnostic techniques including INI-1 IHC and DNA methylation profiling, and age-adapted treatment strategies that prioritize both disease control and functional preservation in a population for whom standard pediatric protocols may be prohibitively toxic.

## Case presentation

**Figure 1:**
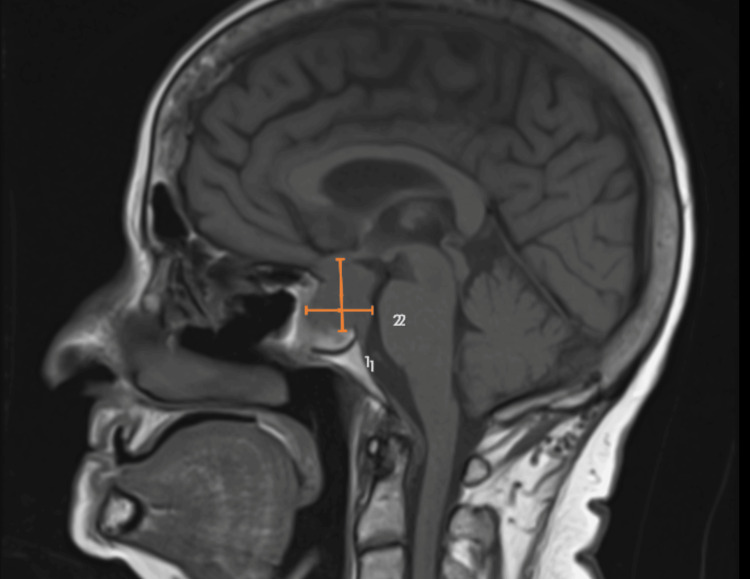
MRI of the brain (T1-weighted post-contrast sagittal image) revealing a pituitary macroadenoma with optic chiasm compression (marked by orange line 1) and probable invasion of the left cavernous sinus (marked by orange line 2). The heterogeneously enhancing sellar mass measures approximately 2.2 × 1.8 cm with suprasellar extension. MRI: magnetic resonance imaging

**Figure 2:**
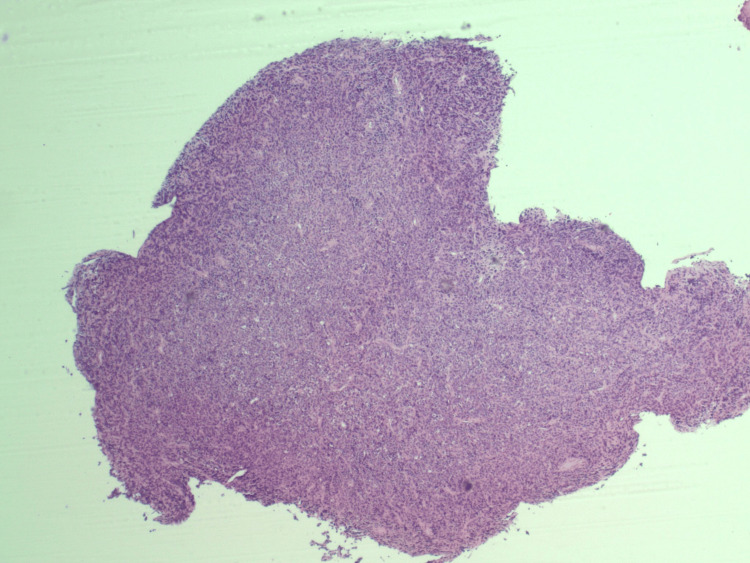
H&E stain at ×40 showing a hypercellular neoplasm with increased nuclear-to-cytoplasmic ratio and infiltrative growth pattern. Note the sheet-like architecture and loss of normal pituitary gland structure, raising concern for an aggressive neoplasm rather than adenoma. H&E: hematoxylin and eosin

**Figure 3:**
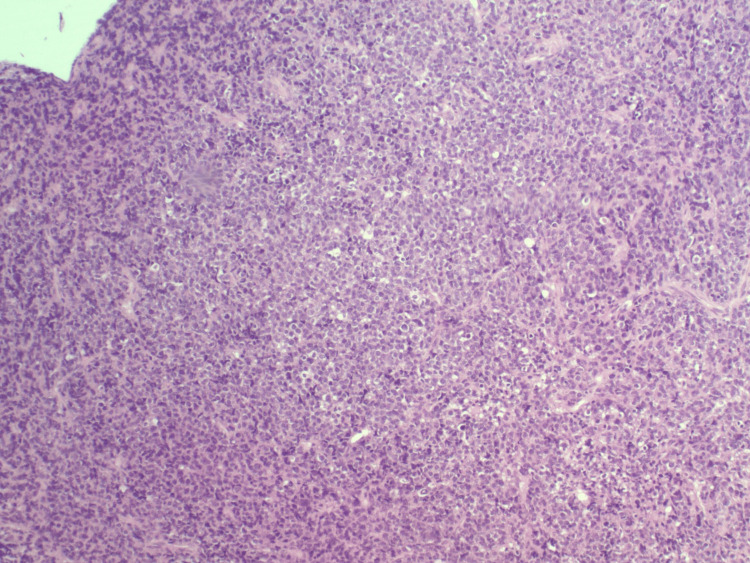
H&E stain at ×100 highlighting sheets of atypical tumor cells with moderate pleomorphism, scattered mitotic activity, and rare apoptotic bodies. Observe the high cellularity and nuclear atypia inconsistent with typical pituitary adenoma. H&E: hematoxylin and eosin

**Figure 4:**
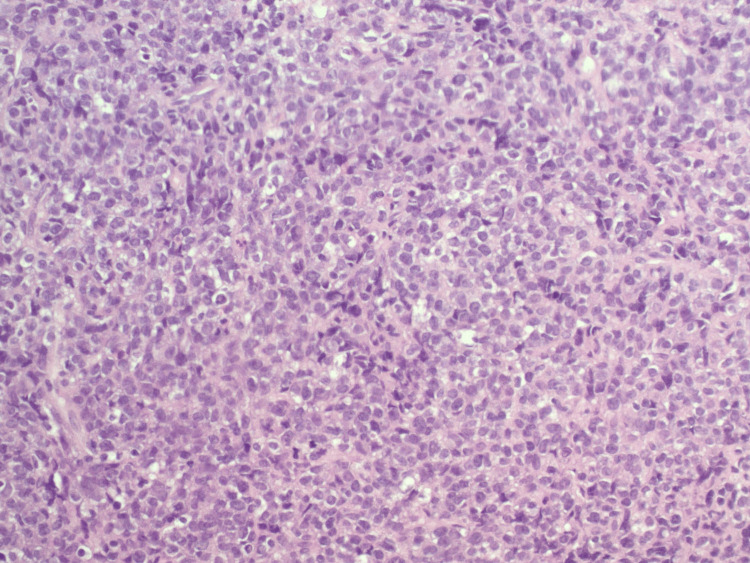
H&E stain at ×200 demonstrating marked nuclear pleomorphism, hyperchromasia, and variably prominent nucleoli. These features, along with high mitotic activity, support the diagnosis of a high-grade malignant neoplasm. H&E: hematoxylin and eosin

**Figure 5:**
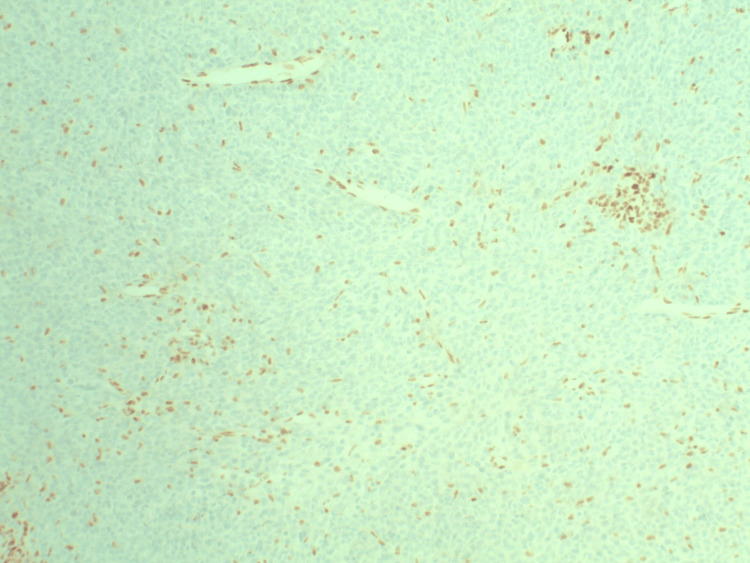
IHC for INI-1 at ×100 magnification demonstrating loss of nuclear staining in tumor cells with preserved staining in endothelial cells (internal control). IHC: immunohistochemistry

A 75-year-old woman with a history of hypertension and hyperlipidemia and an Eastern Cooperative Oncology Group (ECOG) performance status of 1 presented with worsening dizziness following a one-month history of intermittent headaches and visual field defects consistent with chiasmal compression. MRI of the brain imaging revealed a pituitary macroadenoma with optic chiasm compression and probable invasion of the left cavernous sinus, correlating with her presenting symptoms (Figure 1). Imaging included T1-weighted, T2-weighted, and diffusion-weighted sequences. She underwent transsphenoidal hypophysectomy. Histopathologic examination revealed markedly atypical cells with a high nuclear-to-cytoplasmic ratio, nuclear pleomorphism, and an infiltrative growth pattern, findings inconsistent with pituitary adenoma (Figures 2-4). IHC demonstrated loss of INI-1 (SMARCB1) expression with retained SMARCA4, supporting the diagnosis of ATRT (Figure 5). The tumor was positive for vimentin and showed the absence of pituitary hormone markers, excluding pituitary adenoma. The immunohistochemical profile is summarized in Table 1. Although ATRTs are more common in pediatric populations, supratentorial cases, including those in the sellar region, are recognized in adults. Methylome profiling performed at the National Cancer Institute confirmed the diagnosis of ATRT, MYC molecular subtype. Postoperative imaging demonstrated residual sellar mass invasion without evidence of intracranial hemorrhage, midline shift, or hydrocephalus. CSF cytology from lumbar puncture was negative for malignant cells. Representative histopathologic and immunohistochemical findings are shown in Figures 2-5.

**Table 1:** Summary of immunohistochemical staining results confirming the diagnosis of ATRT. Loss of INI-1 expression with retained SMARCA4 is the diagnostic hallmark. ACTH: adrenocorticotropic hormone; GH: growth hormone; PRL: prolactin; TSH: thyroid-stimulating hormone; FSH: follicle-stimulating hormone; LH: luteinizing hormone; ATRT: atypical teratoid/rhabdoid tumor

Marker	Result	Interpretation
INI-1 (SMARCB1)	Loss of nuclear expression	Diagnostic for ATRT; confirms biallelic SMARCB1 inactivation
SMARCA4 (BRG1)	Retained	Rules out SMARCA4-deficient ATRT
Vimentin	Positive	Supports mesenchymal differentiation
Ki-67 proliferation index	>50%	Indicates highly aggressive tumor with rapid growth kinetics
Pituitary hormones (ACTH, GH, PRL, TSH, FSH, LH)	Negative	Excludes pituitary adenoma

A multidisciplinary tumor board recommended craniospinal radiation (20 fractions) with an additional tumor bed boost (13 fractions) and concurrent systemic chemotherapy. Before initiating treatment, the patient was hospitalized for severe, uncontrolled headaches. Comprehensive neuraxis staging was performed with brain MRI, spine MRI, and lumbar puncture with CSF cytology. Repeat brain MRI showed the interval growth of the sellar mass with increased local and regional involvement, consistent with ATRT, but no evidence of new intracranial disease. Spinal MRI revealed degenerative changes and multiple osseous lesions, likely representing benign hemangiomas, with no pathological leptomeningeal enhancement. CSF cytology was negative for malignant cells. Staging computed tomography (CT) scans of the chest, abdomen, and pelvis demonstrated no evidence of metastatic disease. Her symptoms improved with dexamethasone, and she was discharged in stable condition.

She was subsequently readmitted to initiate systemic chemotherapy. On admission, she reported poor oral intake and generalized weakness. Laboratory studies obtained on admission prior to the initiation of systemic chemotherapy revealed baseline thrombocytopenia, renal dysfunction related to dehydration, and metabolic derangements (Table 2). Chemotherapy consisted of standard-dose ICE (ifosfamide, carboplatin, and etoposide) with a 10% dose reduction in ifosfamide due to baseline renal dysfunction. Carboplatin and etoposide were administered at standard doses. Intravenous hydration improved her renal function and clinical status. A repeat lumbar puncture again revealed no malignant cells, with CSF cytology remaining persistently negative across multiple assessments. She completed the first chemotherapy cycle without acute complications and received prophylactic growth factor support with pegfilgrastim. She remained afebrile and asymptomatic, without signs of infection or bleeding during her brief hospitalization.

**Table 2:** Admission laboratory values prior to the initiation of ICE chemotherapy. eGFR: estimated glomerular filtration rate; AST: aspartate aminotransferase; ALT: alanine aminotransferase; MCV: mean corpuscular volume; MCH: mean corpuscular hemoglobin; MCHC: mean corpuscular hemoglobin concentration; RDW: red cell distribution width; MPV: mean platelet volume; RBCs: red blood cells; ICE: ifosfamide, carboplatin, and etoposide

Laboratory test	Value	Reference range	Interpretation
Sodium (mmol/L)	141	135-145	Normal
Potassium (mmol/L)	3.8	3.5-5.0	Normal
Chloride (mmol/L)	115	98-107	High
Bicarbonate (mmol/L)	15	22-29	Low
Anion gap (mmol/L)	11	8-16	Normal
Blood urea nitrogen (mg/dL)	29	7-20	High
Creatinine (mg/dL)	1.19	0.6-1.1	High
eGFR by creatinine (mL/min/1.73 m²)	48	>60	Low
Glucose (mg/dL)	211	70-99 (fasting)	High
Calcium, total (mg/dL)	8.0	8.5-10.5	Low
Phosphorus (mg/dL)	3.7	2.5-4.5	Normal
Alkaline phosphatase (U/L)	77	44-147	Normal
Albumin (g/dL)	2.8	3.4-5.4	Low
Albumin/globulin ratio	1.2	1.1-2.5	Normal
Total protein (g/dL)	5.1	6.0-8.3	Low
Globulin (g/dL)	2.3	2.0-3.5	Normal
Uric acid (mg/dL)	4.8	2.4-6.0	Normal
AST (U/L)	16	10-40	Normal
ALT (U/L)	43	7-35	High
Total bilirubin (mg/dL)	0.5	0.1-1.2	Normal
White blood cells (×10³/µL)	11.2	4.0-10.0	High
RBCs (×10⁶/µL)	4.51	4.2-5.4	Normal
Hemoglobin (g/dL)	14.1	12.0-16.0	Normal
Hematocrit (%)	43.7	36-46	Normal
MCV (fL)	96.9	80-100	Normal
MCH (pg)	31.3	27-33	Normal
MCHC (g/dL)	32.3	32-36	Normal
RDW (%)	13.9	11.5-14.5	Normal
Platelets (×10³/µL)	82	150-450	Low
MPV (fL)	10.8	7.5-11.5	Normal
Nucleated RBCs (%)	0.0	0	Normal
Immature granulocytes (%)	0.7	0-0.5	High
Neutrophils absolute (×10³/µL)	9.68	1.5-8.0	High
Immature granulocytes absolute (×10³/µL)	0.08	0-0.04	High
Lymphocytes absolute (×10³/µL)	0.80	1.0-4.0	Low
Monocytes absolute (×10³/µL)	0.55	0.2-0.8	Normal
Eosinophils absolute (×10³/µL)	0.11	0.0-0.5	Normal
Basophils absolute (×10³/µL)	0.01	0.0-0.2	Normal

**Figure 6:**
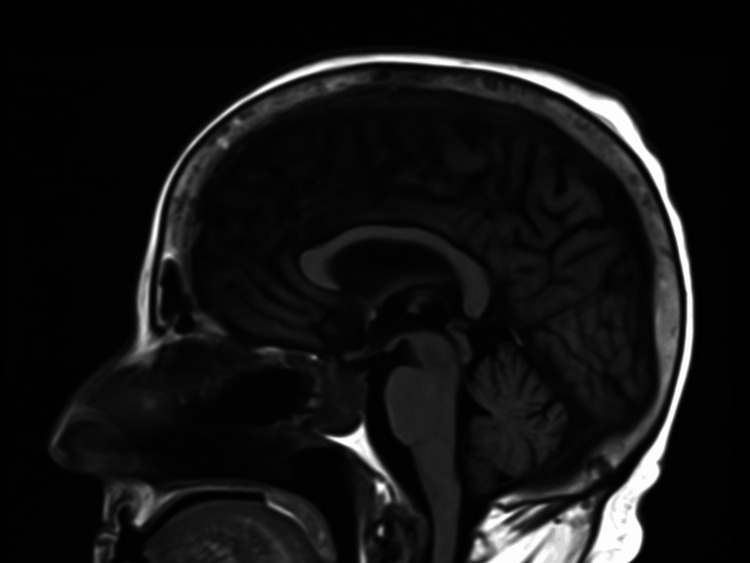
MRI of the brain (T1-weighted post-contrast sagittal image) obtained after the completion of one cycle of ICE chemotherapy and 26 fractions of craniospinal radiation, showing reduced mass effect on the optic chiasm with stable residual sellar disease and no evidence of progression. Interval imaging demonstrates improved decompression compared to baseline (Figure 1). ICE: ifosfamide, carboplatin, and etoposide; MRI: magnetic resonance imaging

Following one cycle of chemotherapy and during concurrent radiation therapy, the patient developed severe metabolic decompensation with critical hyperglycemia, acute kidney injury, and profound pancytopenia (Table 3). Management of treatment-related toxicity required intensive supportive care including red blood cell and platelet transfusions, broad-spectrum antibiotics, granulocyte colony-stimulating factor (G-CSF) support, aggressive hydration, electrolyte repletion, and insulin therapy for steroid-induced hyperglycemia. Radiation therapy was continued during this hospitalization. Interval MRI showed reduced mass effect on the optic chiasm and no evidence of disease progression (Figure 6). Despite these interventions, persistent pancytopenia with critical leukopenia and thrombocytopenia was documented at the time of treatment discontinuation and discharge to rehabilitation (Table 4), reflecting cumulative treatment-related myelosuppression. Due to poor tolerance, radiation therapy was discontinued after 26 sessions, and she was transferred to a rehabilitation facility with plans to resume chemotherapy upon recovery.

**Table 3:** Laboratory values on admission with metabolic derangements. eGFR: estimated glomerular filtration rate; AST: aspartate aminotransferase; ALT: alanine aminotransferase; MCV: mean corpuscular volume; MCH: mean corpuscular hemoglobin; MCHC: mean corpuscular hemoglobin concentration; RDW: red cell distribution width; MPV: mean platelet volume; RBCs: red blood cells

Laboratory test	Value	Reference range	Interpretation
Sodium (mmol/L)	124	135-145	Low
Potassium (mmol/L)	5.4	3.5-5.0	High
Chloride (mmol/L)	97	98-107	Low
Bicarbonate (mmol/L)	10	22-29	Low
Anion gap (mmol/L)	17	8-16	High
Blood urea nitrogen (mg/dL)	35	7-20	High
Creatinine (mg/dL)	1.75	0.6-1.1	High
eGFR by creatinine (mL/min/1.73 m²)	30	>60	Low
Glucose (mg/dL)	551	70-99 (fasting)	Critical high
Calcium, total (mg/dL)	8.4	8.5-10.5	Low
Alkaline phosphatase (U/L)	88	44-147	Normal
Albumin (g/dL)	3.3	3.4-5.4	Low
Albumin/globulin ratio	1.4	1.1-2.5	Normal
Total protein (g/dL)	5.7	6.0-8.3	Low
Globulin (g/dL)	2.4	2.0-3.5	Normal
Lipase (U/L)	120	0-60	High
AST (U/L)	19	10-40	Normal
ALT (U/L)	54	7-35	High
Total bilirubin (mg/dL)	0.4	0.1-1.2	Normal
White blood cells (×10³/µL)	33.7	4.0-10.0	High
RBCs (×10⁶/µL)	3.76	4.2-5.4	Low
Hemoglobin (g/dL)	12.2	12.0-16.0	Normal
Hematocrit (%)	34.1	36-46	Low
MCV (fL)	90.7	80-100	Normal
MCH (pg)	32.4	27-33	Normal
MCHC (g/dL)	35.8	32-36	High
RDW (%)	12.7	11.5-14.5	Normal
Platelets (×10³/µL)	138	150-450	Low
MPV (fL)	10.9	7.5-11.5	Normal
Nucleated RBCs (%)	1.4	0	High
Neutrophils absolute (×10³/µL)	20.89	1.5-8.0	High
Lymphocytes absolute (×10³/µL)	0.34	1.0-4.0	Low
Monocytes absolute (×10³/µL)	1.35	0.2-0.8	High
Eosinophils absolute (×10³/µL)	0.00	0.0-0.5	Normal
Basophils absolute (×10³/µL)	0.00	0.0-0.2	Normal
Metamyelocytes absolute (×10³/µL)	2.70	0	High
Myelocytes absolute (×10³/µL)	7.08	0	High
Promyelocytes absolute (×10³/µL)	1.35	0	High
RBC morphology	Normal	-	Normal
Platelet estimate	Decreased	-	Abnormal

**Table 4:** End-of-admission and discharge laboratory values. The bold values highlight the critical pancytopenia (leukopenia, anemia, and thrombocytopenia) referenced in the preceding paragraph, demonstrating the cumulative treatment-related myelosuppression at the time of treatment discontinuation and discharge to rehabilitation. RBCs: red blood cells; MCV: mean corpuscular volume; MCH: mean corpuscular hemoglobin; MCHC: mean corpuscular hemoglobin concentration; RDW: red cell distribution width; MPV: mean platelet volume

Laboratory test	Value	Reference range	Interpretation
White blood cells (×10³/µL)	1.7	4.0-10.0	Critical low
RBCs (×10⁶/µL)	2.37	4.2-5.4	Low
Hemoglobin (g/dL)	7.8	12.0-16.0	Low
Hematocrit (%)	22.4	36-46	Low
MCV (fL)	94.5	80-100	Normal
MCH (pg)	32.9	27-33	Normal
MCHC (g/dL)	34.8	32-36	Normal
RDW (%)	16.2	11.5-14.5	High
Platelets (×10³/µL)	48	150-450	Low
MPV (fL)	9.4	7.5-11.5	Normal
Nucleated RBCs (%)	1.2	0	High
Immature granulocytes (%)	0.6	0-0.5	High
Neutrophils absolute (×10³/µL)	1.06	1.5-8.0	Low
Immature granulocytes absolute (×10³/µL)	0.01	0-0.04	Normal
Lymphocytes absolute (×10³/µL)	0.47	1.0-4.0	Low
Monocytes absolute (×10³/µL)	0.13	0.2-0.8	Low
Eosinophils absolute (×10³/µL)	0.00	0.0-0.5	Normal
Basophils absolute (×10³/µL)	0.00	0.0-0.2	Normal

Key outcome measures in this case included the preservation of visual function (reduced mass effect on optic chiasm post-treatment), management of endocrine complications related to prolonged corticosteroid use, maintenance of functional status, and minimization of treatment-related hospitalizations. The patient completed 26 of 33 planned radiation fractions and one cycle of chemotherapy before treatment discontinuation due to toxicity, highlighting the challenge of balancing disease control with treatment tolerance in elderly patients with ATRT. Comprehensive genomic profiling revealed no actionable alterations for targeted therapy. Although a second cycle of chemotherapy was planned, her condition deteriorated due to cumulative treatment-related toxicities, and she ultimately passed away at home under hospice care.

## Discussion

The treatment of ATRT relies on a multimodal approach that includes maximal safe surgical resection, chemotherapy, and radiation therapy. GTR, when feasible, is associated with improved survival compared to subtotal resection, and postoperative chemoradiotherapy has been shown to further enhance outcomes [1]. One study reported a median OS of 23.5 months in patients receiving both modalities, emphasizing the importance of an aggressive, multidisciplinary strategy [1]. Protocols such as those from the European Rhabdoid Registry and adaptations of pediatric regimens are frequently used to guide management in adult cases.

Our patient's clinical course aligns with published reports of adult sellar ATRT, which consistently demonstrate poor outcomes despite aggressive multimodal therapy. In a systematic review by Chan et al., the median OS for adult ATRT was 17 months, with particularly dismal outcomes in patients over 60 years of age [3]. Similarly, Zamudio-Coronado et al. reported a median OS of 23.5 months in adult sellar ATRT patients receiving both surgery and chemoradiotherapy, compared to significantly shorter survival with single-modality treatment [1]. Our patient's rapid clinical deterioration despite maximal therapy exemplifies the treatment tolerance challenges unique to elderly patients, whose outcomes are further compromised by age-related frailty, comorbidities, and reduced bone marrow reserve, factors not prominent in pediatric cohorts where two-year event-free survival approaches 78% with similar regimens [2]. The decision to pursue craniospinal irradiation in our elderly patient, extrapolating from pediatric protocols, reflects the absence of age-appropriate guidelines; however, her early treatment-limiting toxicity underscores the need for alternative de-intensification strategies in frail older adults that balance oncologic intent with realistic tolerance thresholds.

In adults, and particularly in elderly patients, there is no consensus standard of care, and treatment strategies are typically extrapolated from pediatric experience. Common chemotherapy backbones include ICE, cisplatin, cyclophosphamide, and high-dose methotrexate [2]. The most recent data from the American Society of Clinical Oncology (ASCO) Publications in the Journal of Clinical Oncology for ATRT in patients three years of age or older shows evidence of prolonged survival with radiation and high-dose alkylator-based chemotherapy [2]. All patients underwent surgical resection; 30 received subsequent chemotherapy. The majority of patients aged three years or older received postoperative craniospinal radiation. Children older than three years typically tolerate these regimens better and achieve higher survival rates, with two-year event-free survival and OS approaching 78% and 89%, respectively [2]. While craniospinal irradiation is better tolerated in adults than in young children, it may still pose substantial risks in the elderly and must be weighed carefully against functional status and comorbidities.

Adult sellar ATRTs exhibit unique patterns of disease spread that distinguish them from pediatric presentations. Notably, pulmonary metastases have been observed and are thought to arise via venous dissemination through the cavernous sinus, particularly in tumors that invade this region. In a case series by Fukuda et al., all patients with pulmonary metastases had evidence of cavernous sinus invasion [13]. Our patient demonstrated bilateral cavernous sinus involvement on initial imaging, raising concern for potential hematogenous dissemination; however, no systemic metastases were identified on staging CT of the chest, abdomen, and pelvis. Invasion into the cavernous sinus may also result in multiple cranial neuropathies, mimicking the presentation of invasive pituitary macroadenoma or carcinoma. Rare presentations involving orbital invasion or spinal drop metastases have also been documented, underlining the need for thorough systemic and neuraxial evaluation in adult patients with ATRT.

Our patient underwent transsphenoidal hypophysectomy followed by craniospinal irradiation and systemic ICE chemotherapy. Her clinical course was complicated by pancytopenia, critical illness myopathy, and steroid-related encephalopathy, which ultimately led to the early discontinuation of radiation and delayed chemotherapy. Given her intolerance to the ICE regimen, alternative therapies such as tazemetostat, an enhancer of zeste homolog 2 (EZH2) inhibitor, were considered. Tazemetostat has shown promise in preclinical models and early-phase trials targeting SMARCB1-deficient tumors, including ATRT, although overall response rates have been modest [15]. In the National Cancer Institute-Children's Oncology Group (NCI-COG) Pediatric Molecular Analysis for Therapy Choice (MATCH) trial (APEC1621C), patients with SMARCB1 or SMARCA4 loss achieved stable disease in select cases, though few objective responses were seen [15].

Other investigational agents, such as ribociclib (a CDK4/6 inhibitor) and alisertib (an Aurora A kinase inhibitor), have also demonstrated limited efficacy, with occasional prolonged disease stability but low overall response rates [16]. Immune checkpoint inhibitors and novel epigenetic modulators are currently being explored in early-phase studies, but their utility in ATRT remains investigational and unproven in larger adult cohorts [16].

Treating ATRT in the elderly presents distinct clinical and ethical challenges. Comorbidities, frailty, and diminished bone marrow reserve often preclude the use of high-dose chemotherapy or stem cell rescue. Tolerance to craniospinal radiation is also reduced, and treatment-related morbidity can be severe. As a result, curative-intent options are often limited, and management must be highly individualized. Multidisciplinary care, including neuro-oncology, radiation oncology, endocrinology, geriatrics, and palliative medicine, is essential to balance oncologic control with functional preservation and quality of life [9,17]. This case exemplifies the delicate therapeutic balance required in elderly patients with ATRT and highlights the need for further research into less toxic, age-adapted treatment strategies.

## Conclusions

ATRT is an exceedingly rare and aggressive malignancy in older adults, particularly when arising in the sellar region. Diagnosis in this population is often delayed due to radiographic resemblance to more common lesions, such as pituitary macroadenomas. Our case illustrates the diagnostic, therapeutic, and supportive care challenges of managing ATRT in an elderly patient, where standard pediatric-based multimodal therapies may be poorly tolerated. Despite surgical resection, craniospinal irradiation, and systemic chemotherapy, our patient experienced significant treatment-related toxicity, ultimately limiting the ability to complete curative-intent therapy.

This case underscores the critical gap between pediatric treatment paradigms, where children over three years achieve two-year event-free survival of 78% and OS of 89% with craniospinal irradiation and high-dose alkylator-based chemotherapy, and the reality of treatment tolerance in elderly patients. Treatment tolerance was markedly limited: our patient completed only 26 of 33 planned craniospinal radiation fractions before discontinuation due to cumulative toxicity and received only one cycle of ICE chemotherapy with dose reduction (10% ifosfamide reduction for renal dysfunction) before clinical deterioration necessitated transition to supportive care. This outcome contrasts sharply with pediatric cohorts where treatment completion rates exceed 85%, illustrating that extrapolation of pediatric protocols to frail older adults often results in excessive treatment burden without corresponding survival benefit, and supports the need for the prospective development of age-attenuated protocols that prioritize quality of life and functional preservation.

Given the lack of established treatment protocols for elderly patients, care must be individualized and multidisciplinary. Emerging targeted therapies, including EZH2 inhibitors and other molecular agents, offer potential future options, though current evidence remains limited. Comprehensive genomic profiling in our patient revealed no actionable alterations amenable to targeted therapy, highlighting that emerging investigational agents such as EZH2 inhibitors, CDK4/6 inhibitors, and Aurora kinase inhibitors, while theoretically promising, remain of uncertain benefit in elderly patients without specific molecular targets and should not delay the consideration of palliative approaches when standard therapy proves intolerable. Key outcome measures in this case, including visual function preservation achieved via surgical decompression, management of treatment-related toxicity requiring prolonged hospitalization and intensive supportive care, and ultimate functional decline necessitating hospice transition, illustrate the unfavorable value proposition of standard pediatric regimens in elderly patients, where treatment-related toxicity can exceed disease burden. This case underscores the urgent need for research into age-adapted treatment approaches and highlights the importance of balancing disease control with quality-of-life considerations in elderly patients with high-grade CNS tumors such as ATRT.
